# Long-term outcome of posterior spinal fusion for the correction of adolescent idiopathic scoliosis

**DOI:** 10.1186/s13013-018-0157-z

**Published:** 2018-08-02

**Authors:** Hasan Ghandhari, Ebrahim Ameri, Farshad Nikouei, Milad Haji Agha Bozorgi, Shoeib Majdi, Mostafa Salehpour

**Affiliations:** Bone and Joint Reconstruction Research Center, Shafa Orthopedic Hospital, Iran University of Medical Sciences, Tehran, Iran

**Keywords:** Adolescent idiopathic scoliosis, Posterior spinal fusion, Complications

## Abstract

**Background:**

Adolescent idiopathic scoliosis (AIS) is the most common form of idiopathic scoliosis, and surgery is considered as one of the therapeutic options. However, it is associated with a variety of irreversible complications, in spite of the benefits it provides. Here, we evaluated the long-term outcome of posterior spinal fusion (PSF) of AIS to shed more light on the consequences of this surgery.

**Methods:**

In a cross-sectional study, a total of 42 AIS patients who underwent PSF surgery were radiographically and clinically inspected for the potential post-operative complications. Radiographic assessments included the device failure, union status, and vertebral tilt below the site of fusion. Clinical outcomes were evaluated using the Oswestry disability index (ODI) and visual analogue scale (VAS).

**Results:**

The mean age of the surgery was 14.4 ± 5.1 years. The mean follow-up of the patients was 5.6 ± 3.2 years. Complete union was observed in all patients, and no device failure was noticed. Pre- and post-operative vertebral tilt below the site of fusion were 11.12° ± 7.92° and 6.21° ± 5.73°, respectively (*p* < 0.001). The mean post-operative ODI was 16.7 ± 9.8. The mean post-operative VAS was 2.1 ± 0.7. ODI value was positively correlated with follow-up periods (*p* = 0.04, *r* = 0.471). New degenerative disc disease (DDD) was observed in 6 out of 37 (16%) patients.

**Conclusion:**

In spite of the efficacy and safety of PSF surgery of AIS, it might result in irreversible complications such as DDD. Moreover, the amount of post-operative disability might increase over the time and should be discussed with the patients.

## Background

Scoliosis is a spinal deformity which refers to deviation of the spine greater than 10° in the coronal plane. Idiopathic scoliosis is the most common type of scoliosis and spinal deformity as well. According to the age of onset, idiopathic scoliosis can be classified as infantile, juvenile, and adolescent [1, 2]. Adolescent idiopathic scoliosis (AIS) is the most common form of idiopathic scoliosis, occurring at the age of 10 years or greater [3].

The treatment options for AIS include observation, bracing, and surgery, and the general goal is to keep curves under 50° at maturity [2, 4]. Available surgical options for the treatment of idiopathic scoliosis include posterior spinal fusion (PSF), anterior spinal fusion (ASF), or a combination of both [5]. PSF remains as the gold standard for the treatment of thoracic and double major curves (most cases). ASF is indicated for thoracolumbar and lumbar cases having a normal sagittal profile. A combination of ASF and PSF could also be used for the management of large curves (> 75°) or stiff curves, young age, and to prevent crankshaft phenomenon [6–9]. The study of Geck et al. on the outcome of surgical management of adolescents with Lenke 5C curves revealed statistically significantly better curve correction, less loss of correction over time, and shorter hospitalization time when treated with a PSF compared with ASF for similar patient populations [10]. Superior outcome of PSF has been reported in other investigations as well [11].

Although the safety and efficacy of both techniques have been demonstrated [5], many patients and surgeons are concerned about the long-term outcome of an extensive fusion in terms of spinal function, the development of degenerative disc disease (DDD), and pain [12]. Weiss et al. reviewed the long-term risks of fusion spinal surgery with respect to the etiology of scoliosis to enable establishing a cost/benefit relation of this intervention. According to their study, average rate of complications was 44% in AIS, ranging from 10 to 78%. They concluded that long-term complications have not yet been fully evaluated and further studies are needed to address this concern adequately [13].

Here, we aimed at evaluating long-term effects of PSF in Iranian AIS patients. To the best of our knowledge, no similar investigation has been earlier performed in Iranian AIS population.

## Methods

In a cross-sectional study, AIS patients who underwent PSF surgery at our center during 2003–2015 were included. Exclusion criteria were (1) congenital, neuromuscular, or infantile scoliosis; (2) history of previous spinal surgery, i.e., discectomy; (3) presence of diseases which might affect the outcome such as rheumatoid arthritis and diabetes mellitus; (4) and unavailable imaging. Accordingly, from a total of 145 AIS patients who were treated with PSF, 52 were identified as eligible for this study. These patients were invited for the evaluation process; from them, 42 patients attended the evaluation session.

Plain standing spinal radiograph of C1–S1 in anteroposterior (AP) and lateral views, along with a lumbosacral MRI without contrast, was taken for radiographic assessments including the evaluation of device failure, union status of fusion site, and vertebral tilt below the site of fusion. Vertebral tilt was measured in both radiographs of before and after surgery using the superior end plate of the inferior disc at the fusion site. Clinical outcome was evaluated using the Oswestry disability index (ODI) and visual analogue scale (VAS), which in both a higher score was equivalent to an inferior outcome.

**Fig. 1:**
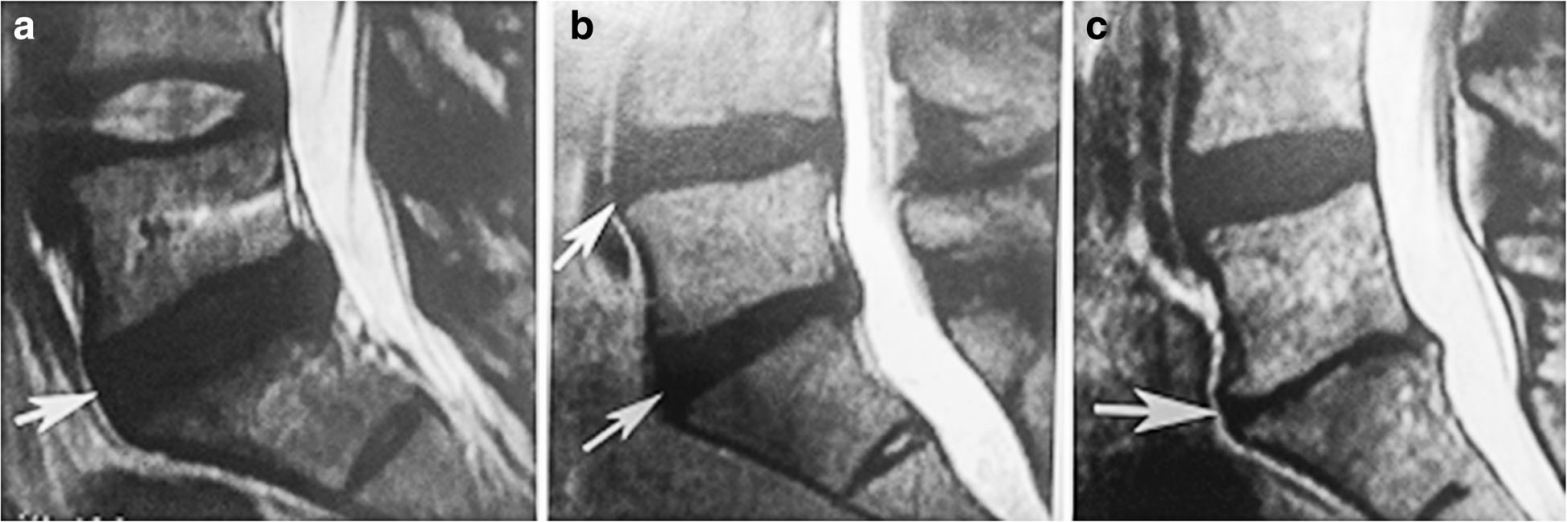
Classification disc degenerative disease using the J. Khanna method: **a** grade 1, **b** grade 2, and **c** grade 3

DDD classification was performed using the J. Khanna classification method. Based on this method, DDD was categorized into three classes. Accordingly, grade 1 was defined as a decrease in disc signal in T2 MRI. Grades 2 and 3 were defined as partial and complete disc collapse, respectively, in MRI imaging [14] (Fig. 1).

### Statistical analysis

Descriptive analysis was performed using mean and standard deviation (SD). *T* test or analysis of variance (ANOVA) was used to compare the mean values between the groups. Pearson’s or Spearman’s correlation coefficient was used to evaluate the potential correlation between the variables. Data analysis was performed using SPSS for windows, version 16. A *p* value of less than 0.05 was considered significant.

## Results

A total of 42 patients with the mean age of 20.5 ± 6.8 years, ranging from 16 to 25 years, were evaluated in this study. The mean age of surgery was 14.4 ± 5.1 years. The mean post-operative follow-up period of patient was 5.6 ± 3.2 years, ranging from 3 to 10 years. The most common level of fusion was L4 followed by L3 and L2. Screw or hook was used as the fusion device. The surgical and demographic characteristics of the patients have been summarized in Table 1.

**Table 1:** The demographic and surgical characteristics of the patients

Characteristic	Mean ± SD or number (%)
Age at the time of study (years)	20.5 ± 6.8
Age at the time of surgery (years)	14.4 ± 5.1
Post-operative follow-up (years)	5.6 ± 3.2
Gender	
• Male	7 (15)
• Female	35 (85)
Distal fusion level	
• L1	1 (2)
• L2	11 (25)
• L3	14 (33)
• L4	15 (35)
• L12	2 (5)
Fusion device	
• Screw	27 (63)
• Hook	15 (27)

Radiographic assessment of the patients confirmed a complete union in all cases. Furthermore, no device failure occurred in any patient of the study population. Mean vertebral tilt below the site of fusion before and after surgery were 11.12° ± 7.92° and 6.21° ± 5.73°, respectively. This difference was statistically significant (*p* < 0.001).

The mean post-operative ODI was 16.7 ± 9.8. The mean post-operative VAS was 2.1 ± 0.7. No significant correlation was observed between the values of VAS and follow-up period (*p* = 0.321, *r* = − 0.157). However, a significant positive correlation was seen between ODI values and follow-up periods (*p* = 0.04, *r* = 0.471), so that a higher ODI value was present in patients with longer follow-up after the surgery. Moreover, ODI values were significantly correlated with post-operative vertebral tilt (*p* = 0.038, *r* = 0.389). No significant association was observed between the fusion level and ODI or VAS (*p* = 0.59 and *p* = 0.44, respectively). The mean ODI and VAS were not significantly different when different devices were used (*p* = 0.6 and *p* = 0.47, respectively).

In total, DDD was present in 5 (15%) patients before the surgery, whereas the disc was normal in the remaining 37 (85%) patients. While at the evaluation session the disc was still normal in 31 (83.8%) out of these 37 patients, grade 1 and grade 2 DDD was developed in 5 (13.5%) and 1 (2.7%) patient, respectively. Most of DDDs (72%) occurred in the first 3–5 years after the surgery. DDD development was not associated with the age of the patients (*p* = 0.12). Occurrence of DDD was also not significantly associated with pre-operative or post-operative vertebral tilt (*p* = 0.3 and *p* = 0.08, respectively). No significant association was also observed between the clinical scores (VAS and ODI) and DDD occurrence (*p* = 0.5 and *p* = 0.53: respectively). Moreover, the level of fusion was not significantly associated with the occurrence of DDD (*p* = 0.87). The DDD occurrence was not associated with the choice of fusion device as well (*p* = 0.14).

The results of surgery have been summarized in Table 2.

**Table 2:** The outcome of the patients following the PSF surgery of AIS

Patients’ characteristics (*n* = 42)	Mean ± SD or number (%)
Pre-operative vertebral tilt	11.12° ± 7.92°
Post-operative vertebral tilt	6.21° ± 5.73°
Post-operative ODI	16.7 ± 9.8
Post-operative VAS	2.1 ± 0.7
New DDD	
• Grade 1	5 (13.5)
• Grade 2	1 (2.7)

*PSF* posterior spinal fusion, *AIS* adolescent idiopathic scoliosis, *DDD* degenerative disc disease, *ODI* Oswestry disability index, *VAS* visual analogue scale

## Discussion

Corrective surgery of AIS can result in several benefits for the affected patients including improvements in esthetics, quality of life, disability, back pain, psychological well-being, and breathing function. It also can stop the progression of curve in adulthood, removing the need for further treatments in adulthood [15]. Based on the study of Ward et al., who compared the outcome of 190 non-operatively treated AIS subjects with 166 operatively treated patients, statistically significant differences in self-image, satisfaction, and total score were found in favor of the operative cohort [16].

On the other hand, AIS surgery still might result in a variety of complications whose long-term impact is poorly understood including neurological damage, loss of normal spinal function, strain on unfused vertebrae, curvature progression, decompensation and increased sagittal deformity, increased torso deformity, delayed paraparesis, and pseudarthrosis [13, 17]. Degenerative disc disease is also considered as one of the late complications of AIS both before and after the surgery, and its association with the severity of pain has been reported [18].

Thus, the surgeons must carefully weigh the potential for improvement against possible operative or post-operative complications. To this aim, further investigations are needed to shed more light on the long-term complications of AIS surgery and help the surgeon to choose the best therapeutic option.

Here, we evaluated the long-term outcome of PSF surgery in 42 AIS patients at a mean follow-up of 5.6 years. Radiographic markers of significant disc degeneration have been reported in nearly 7% of patients 10 years after surgery for AIS. However, the range of this rate varies between studies [19]. According to our study, new DDD was developed in 6 out of 37 (16%) patients with the preoperative normal discs.

Our study showed no association between the development of DDD and clinical findings (ODI and VAS). Similar results were reported in other investigations [20, 21].

While the DDD was more likely to present at the first post-operative 3–5 years in our patients, the clinical outcome was found to be associated with the time past the surgery, so that an inferior outcome was observed in patients with the longer follow-up period. In other words, the observed post-operative disability tended to increase over the time. The study of Upasani et al. also showed an increased pain at 5 years compared with 2 years after AIS surgical treatment [21]. Thus, we suggest surgeons to discuss this long-term complication with their patients prior to the surgery.

According to the study of Green et al., the lower level of fusion was associated with the higher rate and grade of disc degeneration after PSF surgery of AIS [22]. Similar results were reported by Luk et al. [23]. By contrast, Harding et al. found no correlation between disc degeneration and number of fused vertebrae [20]. Our results were in accordance with the results of Harding et al. [20].

Our results revealed a significant association between the preoperative vertebral tilt and post-operative ODI. This finding proposes that a pre-operative higher tilt distal to the site of fusion corresponds to a higher post-operative ODI and could be regarded as a prognostic marker of the surgery.

Our study has some weaknesses which should be pointed out. The small number of cases, caused by the high rate of loss of follow-up, could be regarded as the main weakness of this investigation. This limitation might have adversely affected the statistical power of the study. It also did not allow us to further analyze the data, such as to search an association between the grade of DDD and other variables. Thus, further studies with larger sample size are needed to confirm our results.

## Conclusion

In spite of the benefits it might bring to the affected patients, the surgery of AIS could result in a variety of irreversible complications, including the degenerative change of the discs. Thus, the surgeons must carefully weigh the potential benefits and complications of an AIS surgery prior to the procedure. Moreover, they should inform the patients that some of the observed improvements might reduce over the time.

## Abbreviations

AIS: Adolescent idiopathic scoliosis
ANOVA: Analysis of variance
AP: Anteroposterior
ASF: Anterior spinal fusion
DDD: Degenerative disc disease
MRI: Magnetic resonance imaging
ODI: Oswestry disability index
PSF: Posterior spinal fusion
SD: Standard deviation
VAS: Visual analogue scale
